# Duodenal Metastases From Renal Cell Carcinoma Presented With Melena: Review and Case Report

**Published:** 2017-07-01

**Authors:** Ramesh Omranipour, Habibollah Mahmoud Zadeh, Fershteh Ensani, Samira Yadegari, Seyed Rohollah Miri

**Affiliations:** 1 *Cancer Institute of Tehran University of Medical Science, Tehran, Iran*; 2 *Farabi Hospital, Tehran University of Medical Science, Tehran, Iran*; 3 *Research Center of Cancer, Tehran University of Medical Science, Tehran, Iran*

**Keywords:** Duodenum, Renal cell, carcinoma, Pancreaticoduodenectomy, Melena

## Abstract

Renal cell carcinoma (RCC) metastasis to duodenum is very rare and only a few case reports are available in the literature. We here reported a patient with solitary duodenal metastasis presented with melena six years after right nephrectomy. The patient underwent upper gastrointestinal endoscopy showing ulcerative mass at the second portion of duodenum and biopsy of this mass was consistent with metastatic RCC. Metastasis work up did not find any other site of malignancy, thus Whipple’s operation (Pancreaticoduodenectomy) was performed. In conclusion metastasis from RCC should be considered in mind in patients with history of nephrectomy presenting with gastrointestinal symptoms and a complete evaluation, especially endoscopic examination followed by biopsy, is suggested.

## Introduction

Renal cell carcinoma (RCC) has trend to metastasize many years following surgery. Metastatic sites for RCC include lungs, bones, liver, adrenal glands and brain; however in rare cases, gastrointestinal system can be involved (1).

It can involve any section of the bowel and accounts for 7.1% of all metastatic tumors to the small intestine (2). Duodenal metastasis from RCC is very rare and only few cases have been reported in the literature, also duodenal metastasis generally happens when there is abroad nodal and visceral involvement and clues for metastatic disease elsewhere in the body (2, 3). Generally, RCC metastases occur many years after surgical resection, with recurrences reported up to 16 years after initial surgery (3, 4). Most patients of duodenal metastasis from RCC present with upper gastrointestinal bleeding or obstructive symptoms, and other signs include anemia, melena, fatigue and early satiety. Multiple treatments of solitary RCC metastasis have been discussed. These include a spectrum of surgical and interventional therapy elections that have been shown to enable drastic survival benefits (5, 6, 7). Hence, we reported a patient with solitary duodenal metastasis presented with gastrointestinal bleeding and melena six years after right nephrectomy.

## Case report

A 59-year-old male was presented with black tarry stools and melena. His medical history showed right radical nephrectomy (6 years ago) with the diagnosis of clear renal cell carcinoma treated with adjuvant immunotherapy. He did not have nausea, vomiting or abdominal pain. There was no history of recent use of non-steroidal anti-inflammatory. He had a 20-pack-year history of smoking, but denied any alcohol use. On physical exam, he had orthostatic hypotension and appeared pale. Pertinent physical findings included melanotic stools. Abdominal examination was unremarkable. No signs of chronic liver disease were noted.

**Fig 1:**
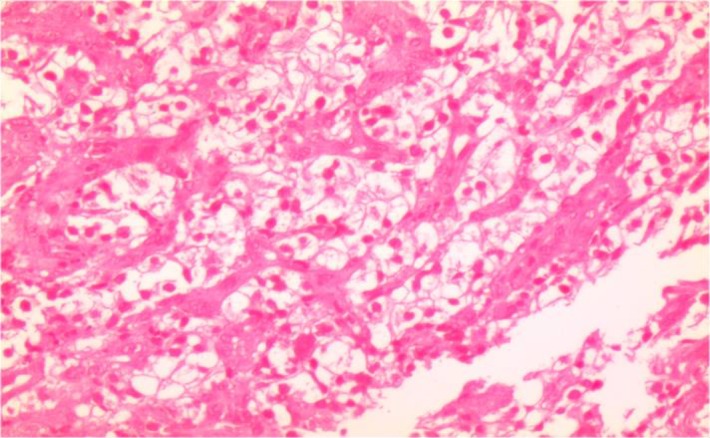
Aggregation of Large Cells With Abundant Cytoplasm

**Fig 2:**
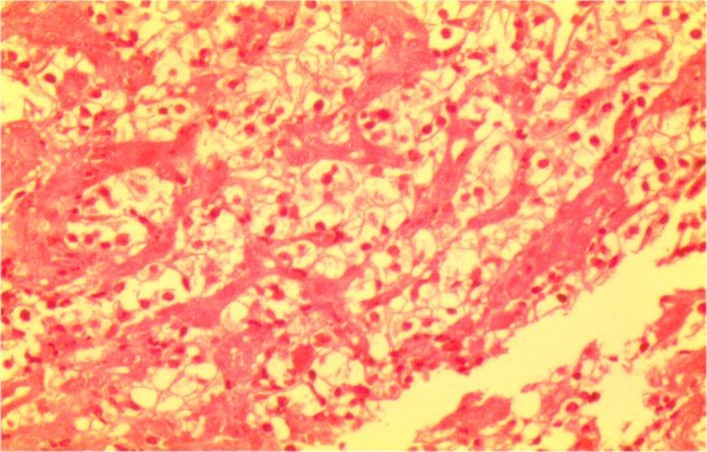
Aggregation of Large Cells With Abundant Cytoplasm

**Fig 3:**
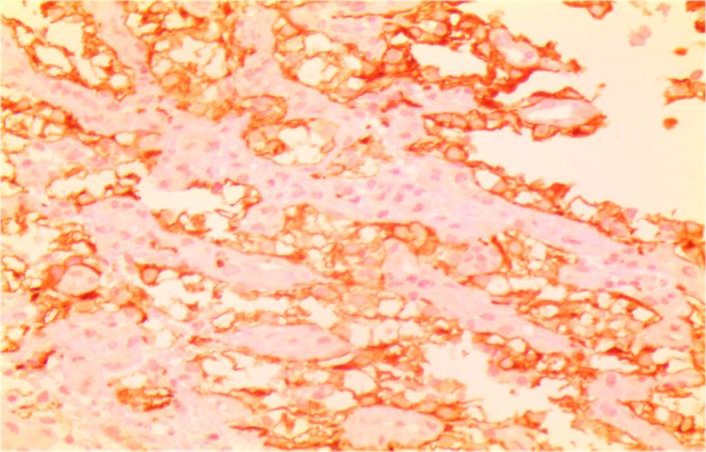
IHC; pan ck, vimentin and CD10 had positive results but CK7,CD68 were negative

**Fig 4:**
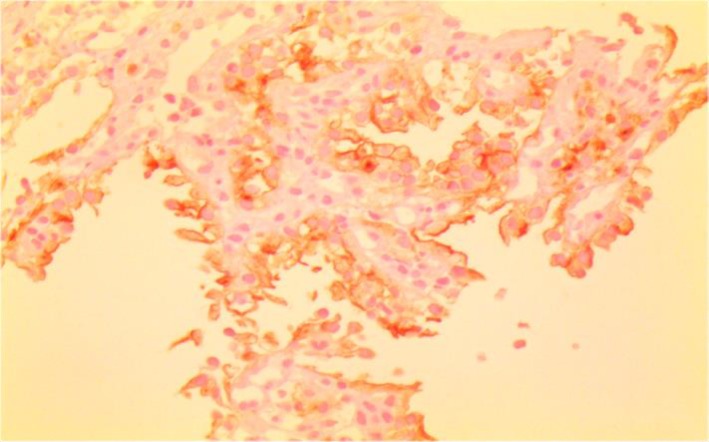
IHC; pan ck, vimentin and CD10 had positive results but CK7,CD68 were negative

**Fig 5:**
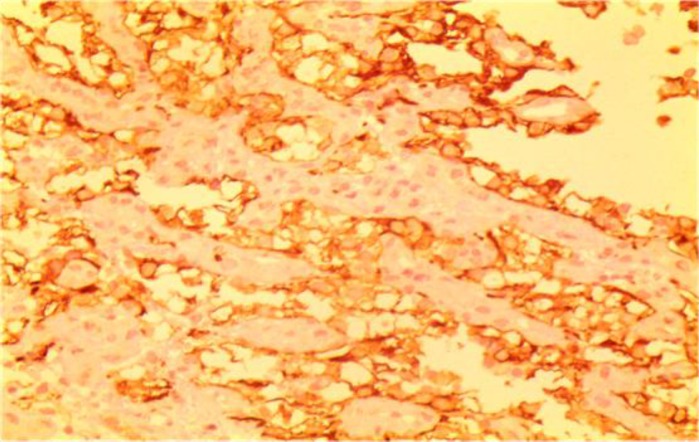
IHC; pan ck, vimentin and CD10 had positive results but CK7 and CD68 were negative

Laboratory investigations on admission included microcytic hypochromic anemia with hemoglobin 9 g/dl, hematocrit 26%, MCV 72 Fl and MCH 21.7 pg/l. Liver enzymes were within the normal range. Esophagogastroduodenoscopy showed a 4×3cm irregular, polypoid, ulcerative mass in the second portion of duodenum. A biopsy was taken and sections from duodenum showed aggregation of large cell with abundant clear cytoplasm (figures 1, 2). Immunohistochemistry had positive results for Pancytokeratin, CD10, vimentin and negative for CD7 and CD68 (Figures 3, 4 and 5). With the pathologic diagnosis of metastatic RCC, metastatic work- up (abdominopelvic, thorax spiral computer tomography, brain MRI and whole body scan) was performed showing no other site for metastases. Computed tomography (CT) scan of the abdomen revealed a large heterogeneous soft tissue mass in the right nephrectomy bed invading the second/proximal third portion of the duodenum (Figure 6), suspicious for recurrent renal cell cancer. Whipple’s operation was performed and the pancreatoduodenal mass was resected. The specimen showed a mass measuring 4×3cm in the duodenum extending to the head of the pancreas. 

The patient was discharged in good condition and followed 6 and 12 months post-operation without any significant complication.

## Discussion

Metastatic malignancies of the small bowel are rare but some tumors may metastasize more frequently than others, such as melanomas, lung cancer, cervical carcinomas, thyroid carcinomas, hepatoma and Merkel cell carcinomas (8,9,10,11). 

RCC has the ability to metastasize to almost any site, but the most common sites are lung (75%), lymph nodes (36%), bone (20%), liver (18%), adrenal glands, kidney, brain, heart, spleen, intestine and skin **(**12)**.** Generally, 4% of RCC metastasize to the GI tract and account for 7.1% of all metastatic tumors to the small intestine (12,13). The duodenum is the very rare site followed by the duodenal bulb **(**9,14,15). A literature review lists all reported cases of renal cell cancer with duodenal metastasis (Table 1). Duodenal metastases ordinarily present as acute or chronic gastrointestinal hemorrhage, duodenal obstruction**,** perforation, duodenal intussusception or as obstructive jaundice (5,13). Diagnosis of duodenal metastases as a cause of GI bleeding is a challenge due to its rarity and thus low index of suspicion for diagnosis. Duodenal lesions may be diagnosed in barium studies or abdominal computer tomography as thickening of the wall or folds in the diseased segment. Endoscopy display non-ulcerative mass, sub-mucosal tumor mass with elevation and ulceration at the apex (volcano lesions) or multiple nodules of multiple sizes with ulceration at the apex (13,16). 

The involvement is commonly the result of direct infiltration, lymphatic, hematogenous or transcoelomic spread (9,15**).** Of note, our case of duodenal metastasis was due to direct infiltration from the recurrent mass in the right nephrectomy bed. In the case of right sided renal cell carcinoma, the contingency of duodenal metastasis is ever higher because of the greater risk of loco-regional invasion (4,5)**. **Patients after nephrectomy for RCC presenting with gastrointestinal symptoms should undergo thorough diagnostic work-up with both endoscopic and radiologic evaluations to assess the extent of metastatic disease (4,5)

In recent articles the longest duration between nephrectomy and duodenal metastasis had been 16 years (15). In this study and some other studies, most of the duodenal metastasis of RCC was after right kidney nephrectomy (15,17).

The standard of treatment for localized metastatic RCC is surgery (18). In previous studies, most patients with duodenal metastasis of RCC were treated with Whipple’s operation; so, there were lucrative surgeries of duodenal saving segmental or wedge resection (19,20). Any type of metastasectomies can increase the survival of patient (21).

The choice of treatment in a case of solitary duodenal RCC metastasis depends on the extent and location of the lesion and therapy must be individually tailored. Procedures such as classic pancreaticoduodenectomy (Whipple procedure) and interventional embolization have been reported (Table 1). Any patient with solitary metastatic RCC to the duodenum should be considered a candidate for complete surgical excision if medically and technically feasible, both for palliation of symptoms and the opportunity for meaningful disease-free survival (22, 23). Therapeutic aims include complete metastatectomy whenever surgically feasible. Any type of metastasectomies can increase the survival of patient (21).

A curative role for pancreaticoduodenectomy in patients with solitary duodenal metastasis has been shown to improve patients’ survival (23, 24, 25). 

For widespread malignancy, treatment is mostly supportive and palliative, in the form of palliative surgery, radiotherapy, chemotherapy or immune-stimulating agents (interleukin-2) (9, 26, 27).

As a conclusion, distant metastasis of RCC can present late with unusual and unpredictable symptoms. In all patients with a history of RCC, gastrointestinal bleeding should be considered as a possible cause of metastasis.
